# Interaction as care in advanced dementia: protocol for a qualitative video-based study of routine care practices

**DOI:** 10.1136/bmjopen-2025-115851

**Published:** 2026-05-04

**Authors:** Anca-Cristina Sterie

**Affiliations:** 1 Service of Palliative and Supportive Care, Lausanne University Hospital, University of Lausanne, Lausanne, Switzerland; 2 Chair of Geriatric Palliative Care, Service of Palliative and Supportive Care and Service of Geriatrics and Geriatric Rehabilitation, Lausanne University Hospital, University of Lausanne, Lausanne, Switzerland; 3 Chair of Palliative Psychology, Service of Palliative and Supportive Care, Lausanne University Hospital, University of Lausanne, Lausanne, Switzerland

**Keywords:** Dementia, Social Interaction, Nursing Homes, QUALITATIVE RESEARCH

## Abstract

**Abstract:**

**Introduction:**

Interaction plays a central role in the delivery of care and in supporting participation and well-being in healthcare. For people living with advanced dementia, cognitive and communicative impairments can restrict participation in everyday care activities while increasing reliance on interaction to obtain support. Although participation is widely promoted in dementia care, empirical evidence on how it is enacted in routine care interactions, particularly in advanced stages of dementia, remains limited. This study aims to examine how healthcare professionals create opportunities for participation for people with advanced dementia during everyday care activities and how these opportunities are taken up, negotiated or resisted in interaction.

**Methods and analysis:**

This is a qualitative multimethod study conducted across six long-term care nursing homes in French-speaking Switzerland. Data collection comprises three phases: (1) ethnographic observations to contextualise care practices and identify target activities; (2) audio-video recordings of naturally occurring interactions between healthcare professionals and people with advanced dementia during routine care activities and (3) collection of complementary sociodemographic, medical and professional data. Approximately 80 residents with advanced dementia and 60 professionals will be involved, yielding an estimated 160–300 recorded interactional sequences. Data will be analysed using conversation analysis, focusing on the sequential and multimodal organisation of interaction. Analysis will examine interactional practices through which professionals create opportunities for participation, residents’ responses to these opportunities and variations across activities, professional roles and care contexts. Findings will inform the identification of interactional competencies relevant for communication skills training.

**Ethics and dissemination:**

Ethical approval has been granted by the Ethics Committee of the Canton of Vaud (205-01373). Informed consent will be obtained from legal representatives of residents and from participating professionals, and assent will be sought from people with advanced dementia. Findings will be disseminated through peer-reviewed publications, academic conferences and feedback to participating institutions and will contribute to the development of communication skills training for healthcare professionals.

**STRENGTHS AND LIMITATIONS OF THIS STUDY**

- The study uses naturalistic audio-video recordings of routine care interactions, allowing fine-grained analysis of verbal and non-verbal practices as they occur in everyday care settings, rather than relying on retrospective accounts or self-report.
- The multisite design across six long-term care nursing homes and the inclusion of multiple professional roles enhances the analytic robustness and transferability of findings across care contexts.
- The use of conversation analysis enables detailed examination of the sequential and multimodal organisation of interaction, which is particularly suited to studying participation among people with advanced dementia who rely heavily on embodied communication.
- The study prioritises the inclusion of people living with advanced dementia through adapted assent procedures; direct involvement of patients or public contributors in study design was not feasible, which may limit incorporation of user perspectives at the planning stage.
- As a qualitative, inductive study, findings will support analytic rather than statistical generalisation, and the study does not aim to quantify effectiveness or establish causal relationships between interactional practices and outcomes.

## Introduction

### Interaction as care

 Care is widely understood as not only crucial for human life but also inherently relational. Philosophers of care emphasise the interdependence between individuals, society and environment, defining care as ‘everything that we do to maintain, continue and repair our ‘world’ so that we can live in it as well as possible’.^1^ In healthcare, care can be defined as ‘a set of relational actions that take place in an institutional context and aim to maintain, improve or restore well-being’.^2^ This definition highlights both the broad scope of healthcare, extending beyond the treatment of illness and its fundamentally relational nature.

Interaction plays a central role in the enactment of care. Most care activities rely, at least partially, on interaction for their accomplishment, and interaction itself can be understood as a form of care. Empirical research shows that interaction affects both quality of care and the well-being of patients and healthcare professionals.^3^ Through interactional processes such as exchanging information, building relationships, enabling autonomy and responding to emotions, care interactions contribute to patient satisfaction, trust, adherence to treatment and shared understanding between patients and clinicians. Even interactional practices often considered peripheral, such as small talk, contribute to establishing social bonds and facilitating instrumental tasks.^4^,^6^ Taken together, these findings underline the importance of considering interaction not as an adjunct to care, but as a core component of healthcare practice.

### Caring dynamics for people living with advanced dementia

Neurocognitive disorders are characterised by progressive impairments in cognitive functioning, affecting domains such as attention, executive function, learning and memory, language, perceptual-motor abilities and social cognition.^7^ These disorders are conceptualised as a cluster and divided into three syndromes: delirium, mild neurocognitive disorder and major neurocognitive disorder, the latter commonly referred to as dementia. In mild neurocognitive disorder, individuals experience cognitive decline while remaining largely independent, whereas in major neurocognitive disorder, cognitive impairment is severe and daily assistance is required. The term ‘advanced dementia’ is used here to refer to this latter stage. The care needs associated with neurocognitive disorders have substantial consequences for healthcare systems, economies and societies more broadly.^8 9^

As dementia progresses, cognitive and communicative abilities required for everyday interaction deteriorate, while dependence on interaction to obtain care and support increases. In advanced stages, symptoms such as apraxia, aphasia and memory decline restrict participation in daily activities and limit self-expression. Nevertheless, quality of life for people living with dementia depends not only on whether care is provided, but on how it is enacted through interaction. Everyday activities such as eating, walking or setting the table can foster connection and delay decline.^10^,^14^ Supporting agency in these contexts requires recognising that interaction itself constitutes a vital form of care.

However, care practices for people with advanced dementia often involve carers ‘doing for’ rather than ‘doing with,’ which may undermine agency and reinforce dependency.^15^,^19^ These dynamics point not merely to deficits in communication, but to how communicative abilities are perceived and negotiated in interaction. In response, care philosophies have increasingly emphasised relational autonomy, foregrounding interdependence and the need to support participation in accordance with a person’s remaining capacities.^1620^,^24^

### Interactional practices in dementia care

Empirical qualitative studies based on recordings of everyday encounters provide valuable insights into how dementia symptoms affect communication in care contexts. This body of research documents linguistic difficulties such as problems with word finding, maintaining reference or recalling events, as well as pragmatic challenges related to turn-taking and meaning-making.^25^,^28^ In advanced stages of dementia, interaction often becomes predominantly non-verbal, relying on gaze, vocalisations or body movement,^29 30^ which can further limit participation in care activities.^17 27 31^

Despite these constraints, people living with dementia continue to display agency through embodied and formulaic forms of communication. Facial expressions and gestures convey intent,^32 33^ while rehearsed or ‘relevant but incorrect’ responses may help preserve social identity.^34^,^36^ Acts such as refusals can also constitute meaningful participation.^37 38^ These interactional complexities pose challenges for carers, who must interpret ambiguous signals and distinguish between overlapping expressions of needs such as anxiety or pain.^39^,^45^ Research shows that participation improves when carers frame activities collaboratively and treat non-verbal behaviours as legitimate contributions to interaction.^3046^,^50^

## Gaps in current research

Despite growing public and scientific attention to dementia, important research gaps remain. People living with dementia, particularly those in advanced stages, are frequently excluded from research due to presumed limitations in interactional or decisional capacity.^51^ This exclusion contributes to marginalisation and limits the empirical basis for developing inclusive care practices. Moreover, much existing research focuses primarily on carers’ perspectives,^52^ with limited first-hand evidence on how people with dementia participate in interaction during everyday care activities.

In addition, empirical studies have mainly examined interactions between people with dementia and relatives^3453^,^55^ or healthcare professionals in clinical encounters,^2756^,^58^ while interactions with other professionals involved in routine care activities such as dressing, washing or grooming remain underexplored. Although participation is widely promoted in policy and institutional discourse,^59^ there is limited empirical evidence on how participation is practically achieved and sustained in everyday care.

Finally, training on interaction with people living with dementia is inconsistently integrated into professional education and is rarely guided by national frameworks. Existing training programmes are often locally developed and unevenly implemented, with approaches such as the validation method^60^ promoted but seldom sustained over time. Given the limited development of research on communication skills training in this field,^61^ there is a pressing need for empirically grounded evidence to inform training, particularly for professionals working with people facing severe communicative challenges.

## Objectives

The primary objective of this study is to capture and analyse interaction practices between healthcare professionals and people with advanced dementia during routine care activities. The study focuses on the interactional strategies through which healthcare professionals vary opportunities for participation and on how people with advanced dementia respond to these opportunities. The secondary objective is to identify interactional practices and competencies that can inform the development of communication skills training for healthcare professionals.

## Methods

### Study design

This study adopts a qualitative multimethod design to investigate interaction practices between healthcare professionals and people living with advanced dementia during routine care activities. The design combines naturalistic observational data with participants’ contextual and professional information in order to capture both how interactions unfold in situ and how they are embedded in institutional care practices. This approach allows for a holistic identification and analysis of interactional phenomena relevant to participation and agency in advanced dementia care.

The study was preceded by a pilot phase that explored the feasibility of employing this methodological approach in the proposed setting.^17^ Findings from the pilot informed refinements to data collection procedures and confirmed the acceptability of observational and video-based methods in long-term care facilities.

We used the Standards for Reporting Qualitative Research (SRQR) reporting guideline^62^ to draft this manuscript, and the SRQR reporting checklist^63^ when editing, included in online supplemental A.

### Phenomena

For the purposes of this study, ‘interaction’ is defined following Erving Goffman as ‘a class of events which occurs during co-presence and by virtue of co-presence’.^64^ Interaction encompasses the glances, gestures, positionings and verbal statements that participants continuously contribute to a situation, whether intentionally or not. This definition includes all verbal and non-verbal manifestations of interaction between two or more participants who orient to one another, and concerns synchronous, face-to-face episodes of exchange.

The concept of interaction is distinguished from that of communication, which focuses primarily on the exchange of information. Interaction is understood more broadly as the means through which participants accomplish activities and coordinate actions in real time. This perspective is particularly relevant for studying care practices involving people with advanced dementia, where embodied and non-verbal conduct often plays a central role.

The study focuses on routine care activities in which people with advanced dementia are involved and to which healthcare professionals contribute. These activities include, but are not limited to, grooming, getting dressed, walking, eating, participating in social activities and attending consultations.

### Participants

Participants include people living with advanced dementia and professionals involved in their care (broadly referred to as ‘healthcare professionals’).

People with advanced dementia are eligible for inclusion if they have a diagnosis of major neurocognitive disorder, as assessed by their healthcare professional using one among three routinely employed instruments: the Clinical Dementia Rating Scale, the Montreal Cognitive Assessment or the Mini Mental State Examination.^65^,^67^ These instruments were selected because they are often used in the context of Swiss nursing homes. Exclusion criteria include psychological or psychiatric conditions that, in the judgement of the healthcare professional, could be exacerbated by participation in the study.

Professionals are eligible if they are working with people living with advanced dementia in their professional environment. They may belong to ‘classic’ healthcare professions (trained nurses, physicians, psychologists, occupational therapists, dentists, nursing assistants) but are not necessarily clinically trained (such as social workers, chaplains, hairdressers, cooks and cooking aids, cleaners, technicians).

No language-based exclusion criteria are applied. This reflects the multilingual context of healthcare institutions in francophone Switzerland, where professionals and residents may interact in languages other than French.^68 69^ This approach also acknowledges evidence that individuals with neurocognitive decline may revert to their native language as dementia progresses.^70^

Overall, the study aims to collect data involving approximately 80 people with advanced dementia and 60 professionals. The potential inclusion of relatives who participate in care activities alongside professionals will be explored during fieldwork and, if relevant, incorporated into the study through an amendment.

### Setting

Data will be collected across six long-term care nursing homes in French-speaking Switzerland. These facilities provide residential accommodation and offer services including personal care, social and recreational activities, meals and medical support. Data collection will be conducted by two PhD candidates (a sociologist and a nurse), under the supervision of the investigator, with a designated study coordinator appointed at each site to support recruitment and coordination.

### Information and consent procedure

The information and consent process is implemented in two phases, reflecting the staged nature of data collection.

For Phase 1 (observational data), oral consent is obtained. As observations are not restricted solely to interactions involving people with advanced dementia, information about the study is provided to all professionals, residents’ legal representatives and relevant relatives.

Written and oral informed consent is obtained from legal representatives of people with advanced dementia. Participants will also be given the option to provide consent for data re-use, for 20 more years after the study’s end. Legal representatives are identified by the study coordinator. Given the close involvement of relatives in residents’ lives, information about the study is also provided to relatives who are not legal representatives.

The consent process for legal representatives proceeds as follows:

1. Prior to Phase 1, legal representatives and, when relevant, relatives identified by the study coordinator are informed of the study via email and invited to attend an oral presentation at the nursing home.
2. Legal representatives of residents with advanced dementia are contacted by the study coordinator to ask whether they agree to being contacted by a PhD candidate for further information.
3. For Phase 2 (video-recorded interactions), legal representatives are contacted by a PhD candidate by telephone to provide detailed information, answer questions and send written consent forms. Legal representatives are given a minimum of 24 hours to return signed consent forms.

Written and oral informed consent is obtained from professionals involved in the care of people with advanced dementia. The procedure includes:

1. A presentation of the study to all professionals at each site prior to data collection.
2. Individual discussions between the PhD candidate and professionals to confirm participation in Phase 2.
3. Reconfirmation of consent prior to each recording session.

People with advanced dementia are informed about the study using adapted language and materials, and their assent is sought in accordance with established qualitative research recommendations.^46 71 72^ This process is informed by consultation with methodological partners experienced in research with this population,^27 37 39 73 74^ project partners and care staff.

### Data collection procedure

Data collection is conducted site by site and comprises three successive components

#### Phase 1: ethnographic observations

Initial ethnographic observations of interactions between professionals and nursing home residents are conducted over a period of 2–4 weeks per site. Observations are carried out by one PhD candidate and focus primarily, though not exclusively, on interactions involving people with advanced dementia. These observations provide contextual understanding of care practices and support the identification of activities to be recorded.^75^ They encompass a broad range of care practices and interactions within the nursing home, including group and social activities as well as activities occurring during both daytime and nighttime periods. The scope of observations is primarily determined by institutional constraints and coordination with the site study coordinator.

At the end of this phase, the PhD candidates, the principal investigator and the study coordinator jointly review preliminary observations and select four routine activities for video recording.

#### Phase 2: audio-video recordings

Audio-video recordings of interactions between professionals and people with advanced dementia are conducted over approximately 1 month per site. Recordings are made using two video cameras, either installed in the environment or carried by the PhD candidate, and aim to capture the natural unfolding of care-related interactions. Recordings focus on the four activities identified during Phase 1. Depending on the size of the nursing home, the study aims to collect between 160 and 300 interactional sequences across sites. All recordings are transcribed verbatim. Specific extracts will be transcribed using a multimodal transcription convention designed for video data, which captures both verbal conduct and embodied actions such as gaze, laughter and movement.^76^,^78^

The choice of care events to record is informed by the observations conducted during Phase 1 and is discussed within the research team and with the site study coordinator. Selection is guided by both feasibility (eg, whether the activity occurs frequently enough to allow multiple recordings) and analytic relevance (eg, activities in which interaction plays a central role or where interactional challenges are commonly observed). For example, if observations indicate that a given activity (such as dining) occurs primarily in group settings, the researchers may also seek opportunities to record less common configurations (eg, in-room dining) in order to capture variation in interactional organisation. Similarly, if a facility implements specific protocols involving particular professionals (eg, occupational therapists or speech therapists) for residents experiencing difficulties such as care refusal or eating problems, these interactions will be actively sought out. During the recording phase, the PhD candidate will aim to capture as many instances as possible of the selected activities in order to document variation in interactional practices across participants and situations.

#### Phase 3: complementary data

Additional data are collected on sociodemographic and medical characteristics of people with advanced dementia, as well as professional background, sociodemographic characteristics and prior communication training of professionals.

### Sample and sampling procedure

The study will collect between 160 and 300 recordings of care activities, in which participate approximately 80 people with advanced dementia and 60 professionals. This number is an estimate based on the prior pilot, oriented to obtain a number of recordings that is sufficient to enable a detailed analysis but also feasible. Resident identification will not be made systematically but in agreement with site coordinators, adapting to what is feasible. All professionals will be considered potential participants, and their participation will be discussed directly with them.

The main objective is to record similar activities across all settings in order to create a large database. Whenever possible, we will also record activities that may be specific to certain institutions, such as hairdressing or dental care (since not all nursing homes employ such professionals). Although the resulting dataset for these activities will be smaller, it will nonetheless help shed light on interactions that have received less attention in previous research. For this secondary objective, purposive sampling will be employed whenever possible (eg, if a resident participating in the study interacts with more ‘unique’ professionals, we will aim to capture these interactions).

### Data processing

Recordings will be made with an Olympus WS-853 pocket recorder and a camera (either a smartphone Google Pixel 5 or a DJI Osmo Nano camera), whose connection to internet is disabled, that will be carried by the PhD candidate in the pocket of the lab coat. Raw data will be uploaded by the PhD candidate to a secure hospital’s platform and accessible only to project staff.

Personal information that appear in the raw data will be coded for verbatim and multimodal transcription. In the transcription, a rendition of the coded content will be available between double parenthesis, for example ((name)). Participants’ identity will be safeguarded to ensure confidentiality; participants will be identified according to a code attributed by the PhD candidate. This coding key will be kept in a list to which only the PhD candidate will have access to, until the end of the study or, if participants give their consent to data re-use, until 20 years after the study’s end.

Raw data will be used for analysis, among the team and occasionally with external experts, who will sign a non-disclosure agreement in advance. No raw data or information about specific participants will be shared with study sites.

When data will be shared with larger audiences (eg, during conferences or papers), it will be adequately coded to ensure that participants’ recognition is impossible. Personal information will be removed from transcripts and audio files, and if video is presented, faces and environments will be blurred.

### Theoretical framework

Ethnographic observations and audio-video recordings are analysed using conversation analysis (CA). CA involves detailed, sequential analysis of naturally occurring interactions and focuses on how participants coordinate actions to accomplish both routine and interactionally challenging tasks, such as initiating activities, making requests or managing resistance.^79^

Analysis is conducted both independently and collaboratively by the research team through regular data sessions. These sessions enable close examination of interactional sequences and support analytic reflexivity and consistency. The analysis attends to two fundamental dimensions of interaction: temporality and sequentiality, examining how actions are produced and responded to over time.^80^

While CA is primarily qualitative and inductive, it also allows for quantitative exploration through the identification of recurrent practices, the categorisation of interactional patterns, and the examination of associations between interactional practices and contextual or sociodemographic variables.^81^ Recent literature highlights the growing relevance of CA for studying communication involving people with cognitive and communicative impairments.^82 83^

### Analysis

The study does not evaluate a clinical intervention; instead, it examines interactional strategies used by professionals during routine care activities. These strategies constitute the primary exposures of interest. The primary outcome is the organisation of opportunities for participation for people with advanced dementia and the ways these opportunities are taken up, negotiated, resisted or not responded to during ongoing care. Opportunities for participation will be operationalised as professional actions (verbal and/or embodied) that make resident participation relevantly possible (eg, invitations to act, requests that allocate a resident turn, offers involving choice, embodied scaffolding that enables action). Resident responses will be analysed sequentially and categorised descriptively (eg, uptake, negotiation, refusal, withdrawal/no response, alternative action) based on the multimodal conduct displayed in the recorded episodes.

Comparisons will be conducted across activity types (eg, grooming, dressing, eating), professional roles, sites, and contextual features such as language concordance/discordance and prior communication training. Analysis will follow established CA procedures, including the development of collections of recurrent practices, detailed examination of sequential environments, and examination of deviant cases. Where appropriate, findings will be complemented by descriptive quantification of recurrent practices and response trajectories to support comparison across contexts.

### Risk management

Video recording is generally acceptable in healthcare settings,^84^ although working with people living with advanced dementia may present additional challenges related to gatekeeping by staff and participants’ vulnerability.^85^ Risk management strategies draw on best practices identified in the literature,^77^ the principal investigator’s prior experience with video recording in nursing homes^17^ and ongoing consultation with methodological and stakeholder partners.

To minimise potential distress, recordings are discontinued immediately if signs of unease are observed in people with advanced dementia. The initial observation phase allows the PhD candidates to build familiarity with care teams, fostering trust and facilitating participation. Given the emotional and ethical demands of fieldwork, PhD candidates receive structured support through integration into an existing research team, participation in monthly reflexivity workshops and regular feedback sessions with the principal investigator during data collection.

### Methodological rigour and trustworthiness

To enhance methodological rigour and transparency, several strategies are implemented throughout the study. First, the use of naturally occurring audio-video recordings allows for repeated, fine-grained analysis of interactional practices and reduces reliance on retrospective accounts or self-report. Second, data analysis is conducted both independently and collaboratively through regular data sessions, among the investigator (author), the two PhD candidates and a postdoctoral researcher, enabling reflexive discussion of analytic interpretations and promoting consistency in analytic decisions, while also incorporating consultations with three external collaborators who bring additional expertise in the topic and methodology. Regularly, representatives of healthcare professionals and relatives of people with advanced dementia will be invited to join data sessions.

The researchers involved in data collection bring complementary disciplinary backgrounds. One PhD candidate is trained in sociology, while the other is a registered nurse with clinical experience in healthcare settings. These backgrounds support both sensitivity to the clinical organisation of care practices and the interactional focus of the study. At the same time, the potential influence of researchers’ disciplinary perspectives on the identification of interactions to be recorded is addressed through the use of shared observation procedures and through regular team discussions during data collection.

Analytic transparency is supported through systematic documentation of analytic steps, including the identification of focal interactional phenomena, the development of analytic collections and the comparison of cases across participants, activities and sites. Deviant cases are actively sought and examined to refine analytic claims and avoid idealisation of interactional practices. Analytic decisions and emerging findings will be documented throughout the study to ensure transparency and support reflexive engagement with the data.

Reflexivity is maintained throughout the research process through regular team discussions and reflexivity workshops, which address the researchers’ positionality and assumptions in relation to the data.

Although the study does not aim for statistical generalisation, analytic generalisability is sought through the identification of recurrent interactional patterns across multiple settings, activities and participants. The inclusion of multiple sites and professional roles further strengthens the robustness of the findings.

## Patient and public involvement

People living with advanced dementia were not directly involved in the design of the study. Direct involvement in study design was considered but deemed not feasible due to the advanced stage of cognitive and communicative impairments of the target population, which limits sustained participation in conventional research planning activities.

Nevertheless, the study is explicitly designed to focus on the perspectives and participation of people living with advanced dementia through the analysis of naturally occurring interactions in everyday care activities. Rather than relying on proxy reports or retrospective accounts, the methodology prioritises first-hand, in situ evidence of how people with advanced dementia engage, respond and exercise agency in interaction. This approach is consistent with recommendations for inclusive qualitative research with populations experiencing severe communicative challenges.^29 71 72^

In addition, the study design and consent procedures were informed by consultation with methodological partners and researchers experienced in conducting interactional research with people living with dementia and other vulnerable populations as well as through ongoing dialogue with care professionals involved in dementia care. Findings from the study will be disseminated to participating institutions and will inform the development of communication skills training for healthcare professionals, with the aim of improving everyday care practices for people living with advanced dementia.

## Ethics and dissemination

The study is approved by the Ethics Committee of the Canton of Vaud (2025-01373). This approval stands for the study’s methodology and includes contracts within three initial nursing homes. Approval for the remaining sites will be requested by the submission of amendments, 6 months before data collection on each site.

## Supplementary material

10.1136/bmjopen-2025-115851 online supplemental file 1
