# Supplementary material for: Interaction as care in advanced dementia: protocol for a qualitative video-based study of routine care practices
Source: BMJ Open. 2026 May 4;16(5):e115851. doi: 10.1136/bmjopen-2025-115851 (PMC13141121; doi:10.1136/bmjopen-2025-115851)
Supplement: online supplemental file 1 [file bmjopen-16-5-s001.docx]

The SRQR reporting checklist

For checking that qualitative health research articles can be understood and used by everyone

| 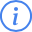 Note |
| --- |
| If you have not used a reporting guideline before, read about [how and why to use them](https:/resources.equator-network.org/about/reporting-guidelines.html) and check whether SRQR is the [most applicable reporting guideline](https:/resources.equator-network.org/reporting-guidelines/srqr/index.html?#applicability) for your work.  Reporting guidelines are most useful when used early in research. When writing a manuscript or application, consider using the [Full Guidance](https:/resources.equator-network.org/reporting-guidelines/srqr/index.html) where you’ll see explanations and examples for each item.  After writing, demonstrate adherence by completing this checklist:   1. Specify where each item is described (see [Note 1](#sec-specify)). 2. Cite this checklist (See [Note 2](#sec-cite)). 3. Include your completed checklist as a supplement when submitting to a journal so that future readers can use it to find information. |

|  | Item Description | Location (or reason for not reporting) |
| --- | --- | --- |
| **Title & Abstract** |  |  |
| Title | Describe the nature and topic of the study. Identify the study as qualitative or indicate the approach or data collection methods. | Yes, see title |
| Abstract | Summarise the key elements of the study using the abstract format of the intended publication. | Yes, see p. 2 |
| **Introduction** |  |  |
| Problem Formulation | Describe the problem/phenomenon studied, its significance, relevant theory and empirical work, and gaps in current knowledge. | Yes, see “Gaps in current research” |
| Purpose or research question | Describe the purpose of the study and specific objectives or questions. | Yes, see “Objectives” |
| **Methods** |  |  |
| Qualitative approach and research paradigm | Describe your qualitative approach, your guiding theory (if appropriate), and research paradigm, and reasons for your choices. | Yes, see “Study design” |
| Researcher characteristics and reflexivity | Describe how researchers’ characteristics may influence the research, including personal attributes, qualifications/experience, relationship with participants, assumptions, and/or presuppositions; potential or actual interaction between researchers’ characteristics and the research questions, approach, methods, results and/or transferability. | Not reporting – researchers haven’t been identified yet |
| Context | Describe the setting/site(s) in which the study was conducted, why it was selected, and any other salient contextual factors that may influence the study. | Yes, see “Setting” |
| Sampling strategy | Describe how and why research participants, documents, or events were selected; criteria for deciding when no further sampling was necessary, and the rationale for those criteria. | Yes, see “Sample and sampling procedure” |
| Ethical issues pertaining to human subjects | Describe any approval by an appropriate ethics review board and participant consent, or explain any lack thereof. Describe any other confidentiality and data security issues. | Yes, see “Information and consent procedure” and “Ethics and dissemination” |
| Data collection methods | Describe the types of data collected; details of data collection procedures including (as appropriate) start and stop dates of data collection and analysis, iterative process, triangulation of sources/methods, and modification of procedures in response to evolving study findings. Describe your rationale for these choices. | Yes, see “Data collection procedure” |
| Data collection instruments and technologies | Describe any instruments (e.g., interview guides, questionnaires) and devices (e.g., audio recorders) used for data collection; describe if/how the instrument(s) changed over the course of the study. | Yes, see “Data collection procedure” |
| Units of study | Describe the number and relevant characteristics of participants, documents, or events included in the study. Describe the level of participation. | Yes, see “Setting” |
| Data processing | Describe the methods for processing data prior to and during analysis, including transcription, data entry, data management and security, verification of data integrity, data coding, and anonymisation / deidentification of excerpts. | Yes, see “Data processing” |
| Data analysis | Describe the process by which inferences, themes, etc. were identified and developed, including the researchers involved in data analysis; usually references a specific paradigm or approach. Describe why you chose this process. | Yes, see “Theoretical framework” and “Analysis” |
| Techniques to enhance trustworthiness | Describe any techniques to enhance trustworthiness and credibility of data analysis,(e.g., member checking, triangulation, audit trail). Describe why you chose these techniques. | Yes, see “Methodological rigour and trustworthiness” |
| **Results** |  |  |
| Synthesis and interpretation | Describe the main findings (e.g., interpretations, inferences, and themes); might include development of a theory or model, or integration with prior research or theory. | Not relevant – data is unavailable yet |
| Links to empirical data | Provide evidence (e.g., quotes, field notes, text excerpts, photographs) to substantiate analytic findings. | Not relevant – data is unavailable yet |
| **Discussion** |  |  |
| Integration with prior work, implications, transferability, and contribution(s) to the field | Summarize the main findings, explain how findings and conclusions connect to, support, elaborate on, or challenge conclusions of earlier scholarship; discuss the scope of application/generalizability; identify unique contribution(s) to scholarship in a discipline or field. | Not relevant – data is unavailable yet |
| Limitations | Discuss the trustworthiness and limitations of findings | Yes, see “Methodological rigour and trustworthiness” |
| **Other** |  |  |
| Conflicts of interest | Describe any potential sources of influence or perceived influence on study conduct and conclusions. Describe how these were managed. | Yes, see “**Competing interests statement”** |
| Funding | Describe sources of funding and other support. Describe the role of funders in data collection, interpretation, and reporting. | Yes, see “**Funding statement”** |
