# Supplementary material for: Interaction as care in advanced dementia: protocol for a qualitative video-based study of routine care practices
Source: BMJ Open. 2026 May 4;16(5):e115851. doi: 10.1136/bmjopen-2025-115851 (PMC13141121; doi:10.1136/bmjopen-2025-115851)
Supplement: Supplementary file 2 [file bmjopen-2025-115851.reviewer_comments.pdf]

PEER REVIEW HISTORY

BMJ Open publishes all reviews undertaken for accepted manuscripts. Reviewers are asked to complete a checklist review form and are provided with free text boxes to elaborate on their assessment. These free text comments are reproduced below.

ARTICLE DETAILS

Title (Provisional)

Interaction as care in advanced dementia: protocol for a qualitative video-based study of routine care practices

Authors

Sterie, Anca-Cristina

VERSION 1 - REVIEW

**Reviewer**	**1**
**Name**	**McConnell , Eleanor**
**Affiliation**	**Duke University, School of Nursing**
**Date**	**06-Feb-2026**
**COI**	**None**

This protocol paper is very well written, and addresses a very important and relatively under-studied aspect of care for people living with major neurocognitive disorders. The motivation for the study is clear, as are the aims, methods, and approaches to conduct of ethical research in a very challenging care setting. The methods are rigorous and are likely to yield important insights into care interactions that could improve quality of care and wellbeing among people living with advanced state dementia. I have only minor suggestions for clarification.

p.7, lines 53-55, the authors state that "people with advanced dementia are eligible for inclusion if they have been assessed by their health care professionals using at least two routinely employed instruments" and then they list the Montreal Cognitive Assessment (MoCA), the Mini-Mental State Exam), and the Clinical Dementia Rating (CDR) Scale. If the rationale for using two is to verify functional impairment, I would have expected that they would require a cognitive test (MoCA OR MMSE) and a CDR. If this is not their intent, it would be good to clarify the rationale for two tests.

Throughout the paper, the authors refer to "health care professionals" who may or may not be clinically trained. It would be helpful for them to specify these professionals, and how they will prioritize (or perhaps stratify) their approach to data collection. For example, it is likely that the professionals will include professionally trained nurses, direct care providers

such as nursing assistants, as well as social workers, physicians, and recreation or occupational therapists, and chaplains or other pastoral care providers. Given that they have specified activities of interest in the protocol (grooming, dressing, eating), the authors may believe the health care professions to be observed are implied, but for transparency, I would recommend listing the potential health care professionals, and whether they plan some type of purposive sampling to ensure they capture the full range of possible interactional styles.

. Please include a description of the professional or discipline background of the PhD candidates who will be collecting data. Will they be clinically trained? It seems to me that professional/disciplinary background of data collector might influence decision-making about what care interactions to record.

Will the data collector observe care routines during daytime hours only, or will observations occur during evening and night-time care? As sleep disturbance is a common symptom experienced by people living with advanced dementia, focusing on day-time activities only is an important limitation. Will the observations focus on direct care, or on group or social activities as well?

I recognize that Phase 1 observations will influence the choice of care interactions to be observed, and likely the health care professionals. However, I wonder if the authors could offer some examples, perhaps from their pilot work, about how they select care episodes. For example, if, during Phase 1, they learn that dining occurs primarily in a group setting, would they purposively seek out 'in-room' dining to observe for differences in interaction approaches? Similarly, if a nursing home has specific protocols for involving specific professionals (occupational therapists, speech pathologists) in care of people who are having a high frequency of care refusal or difficulty with eating, will those interactions be sought out? As written, it is very unclear which care events and professionals will be prioritized.

VERSION 1 - AUTHOR RESPONSE

Dear Reviewer 1,
Thank you for your careful reading of the manuscript and for your thoughtful and constructive comments. Your suggestions have helped improve the clarity and quality of the paper.
Below I provide a detailed, point-by-point response to each comment and indicate where revisions have been made in the manuscript.
Best regards,
Author.

Comment 1. Clarify rationale behind at least two cognitive assessment tools. p.7, lines 53-55, the authors state that "people with advanced dementia are eligible for inclusion if they have been assessed by their health care professionals using at least two routinely employed instruments" and then they list the Montreal Cognitive Assessment (MoCA), the Mini-Mental State Exam), and the Clinical Dementia Rating (CDR) Scale. If the rationale for using two is

to verify functional impairment, I would have expected that they would require a cognitive test (MoCA OR MMSE) and a CDR. If this is not their intent, it would be good to clarify the rationale for two tests.
Response: Thank you for identifying this aspect to be further explained. Since the submission of this paper we have started negotiations with nursing homes and actually very few residents have two of these assessments. In order to render identification and recruitment more feasible, it will be possible to use any of the three instruments. Here below is the newly inserted paragraph:
"Participants:
People with advanced dementia are eligible for inclusion if they have a diagnosis of major neurocognitive disorder, as assessed by their healthcare professional using one among three routinely employed instruments: the Clinical Dementia Rating Scale, the Montreal Cognitive Assessment, or the Mini Mental State Examination (64-66). These instruments were selected because they are often used in the context of Swiss nursing homes."
Comment 2. Define more precisely which health care professionals' interactions will be the focus of the observations. Throughout the paper, the authors refer to "health care professionals" who may or may not be clinically trained. It would be helpful for them to specify these professionals, and how they will prioritize (or perhaps stratify) their approach to data collection. For example, it is likely that the professionals will include professionally trained nurses, direct care providers such as nursing assistants, as well as social workers, physicians, and recreation or occupational therapists, and chaplains or other pastoral care providers. Given that they have specified activities of interest in the protocol (grooming, dressing, eating), the authors may believe the health care professions to be observed are implied, but for transparency, I would recommend listing the potential health care professionals, and whether they plan some type of purposive sampling to ensure they capture the full range of possible interactional styles.
Response: Thank you for signalling this; the diversity of professionals is indeed one of the original aspects of the study and it should be better emphasised. Here below are the newly inserted paragraphs:
"Participants
Professionals are eligible if they are working with people living with advanced dementia in their professional environment. They may belong to "classic" healthcare professions (trained nurses, physicians, psychologists, occupational therapists, dentists, nursing assistants) but are not necessarily clinically trained (such as social workers, chaplains, hairdressers, cooks and cooking aids, cleaners, technicians)."
(…)
"Sample and sampling procedure
All professionals will be considered potential participants, and their participation will be discussed directly with them.
The main objective is to record similar activities across all settings in order to create a large database. Whenever possible, we will also record activities that may be specific to certain institutions, such as hairdressing or dental care (since not all nursing homes employ such professionals). Although the resulting dataset for these activities will be smaller, it will nonetheless help shed light on interactions that have received less attention in previous research. For this secondary objective, purposive sampling will be employed whenever possible (e.g., if a resident participating in the study interacts with more "unique" professionals, we will aim to capture these interactions)."

Comment 3. Clarify Background of Data Collection personnel. Please include a description of the professional or discipline background of the PhD candidates who will be collecting data. Will they be clinically trained? It seems to me that professional/disciplinary background of data collector might influence decision-making about what care interactions to record.
Response: Thank you for this helpful comment. I have added a description of the disciplinary and professional backgrounds of the two PhD candidates responsible for data collection in the "Setting", as well as a brief discussion of their backgrounds may shape observational decisions. Here below are the newly inserted paragraphs:
"Setting:
Data collection will be conducted by two PhD candidates (a sociologist and a nurse), (…)"
(…)
"Methodological rigour and trustworthiness
The researchers involved in data collection bring complementary disciplinary backgrounds. One PhD candidate is trained in sociology, while the other is a registered nurse with clinical experience in healthcare settings. These backgrounds support both sensitivity to the clinical organisation of care practices and the interactional focus of the study. At the same time, the potential influence of researchers' disciplinary perspectives on the identification of interactions to be recorded is addressed through the use of shared observation procedures and through regular team discussions during data collection."

Comment 4. Expand on Phase 1 Approaches to ethnographic observations. Will the data collector observe care routines during daytime hours only, or will observations occur during evening and night-time care? As sleep disturbance is a common symptom experienced by people living with advanced dementia, focusing on day-time activities only is an important limitation. Will the observations focus on direct care, or on group or social activities as well?
Response: Thank you for this comment, it is indeed the intent to observe night-time activities, though this is something that is negotiated with each nursing home individually. Here below is the newly inserted paragraph:
"Phase 1: Ethnographic observations
(…) They encompass a broad range of care practices and interactions within the nursing home, including group and social activities as well as activities occurring during both daytime and nighttime periods. The scope of observations is primarily determined by institutional constraints and coordination with the site study coordinator."

Comment 5. Expand on Phase 2 approach to selecting care events to audio and video record. I recognize that Phase 1 observations will influence the choice of care interactions to be observed, and likely the health care professionals. However, I wonder if the authors could offer some examples, perhaps from their pilot work, about how they select care episodes. For example, if, during Phase 1, they learn that dining occurs primarily in a group setting, would they purposively seek out 'in-room' dining to observe for differences in interaction approaches? Similarly, if a nursing home has specific protocols for involving specific professionals (occupational therapists, speech pathologists) in care of people who are having a high frequency of care refusal or difficulty with eating, will those interactions be sought out? As written, it is very unclear which care events and professionals will be prioritized.
Response: Thank you for the comment. The choice of activities to be recorded is so very dependent on the institution that it is very difficult to make such suppositions. This is mostly

due to the fact that certain institutions might be more "open" in regard to what can be recorded and others very strict, so we wouldn't want to be that specific in the protocol. I have used your suggestions to clarify this aspect a bit further, hopefully sufficiently. Please find below the newly inserted paragraph:

"Phase 2: Audio-video recordings

(…) The choice of care events to record is informed by the observations conducted during Phase 1 and is discussed within the research team and with the site study coordinator. Selection is guided by both feasibility (e.g., whether the activity occurs frequently enough to allow multiple recordings) and analytic relevance (e.g., activities in which interaction plays a central role or where interactional challenges are commonly observed). For example, if observations indicate that a given activity (such as dining) occurs primarily in group settings, the researchers may also seek opportunities to record less common configurations (e.g., in-room dining) in order to capture variation in interactional organisation. Similarly, if a facility implements specific protocols involving particular professionals (e.g., occupational therapists or speech therapists) for residents experiencing difficulties such as care refusal or eating problems, these interactions will be actively sought out. During the recording phase, the PhD candidate will aim to capture as many instances as possible of the selected activities in order to document variation in interactional practices across participants and situations."
